# Comparison of mitral regurgitation severity assessments based on magnetic resonance imaging and echocardiography in patients with hypertrophic cardiomyopathy

**DOI:** 10.1038/s41598-021-99446-y

**Published:** 2021-10-06

**Authors:** Mateusz Śpiewak, Mariusz Kłopotowski, Ewa Kowalik, Łukasz Mazurkiewicz, Katarzyna Kożuch, Joanna Petryka-Mazurkiewicz, Barbara Miłosz-Wieczorek, Adam Witkowski, Anna Klisiewicz, Magdalena Marczak

**Affiliations:** 1 Magnetic Resonance Unit, National Institute of Cardiology, ul. Alpejska 42, 04-628 Warsaw, Poland; 2 Department of Interventional Cardiology and Angiology, National Institute of Cardiology, Warsaw, Poland; 3 Department of Congenital Heart Diseases, National Institute of Cardiology, Warsaw, Poland; 4 Department of Cardiomyopathies, National Institute of Cardiology, Warsaw, Poland; 5 Department of Coronary Artery Disease and Structural Heart Diseases, National Institute of Cardiology, Warsaw, Poland

**Keywords:** Cardiology, Medical research

## Abstract

Mitral regurgitation (MR), which is one of the factors responsible for heart failure symptoms and the development of atrial fibrillation, is an important feature of hypertrophic cardiomyopathy (HCM), and its presence affects which treatment options are chosen. Although cardiac magnetic resonance imaging (MRI) is considered the reference standard for assessing the regurgitant volume (RV) and fraction (RF), echocardiography is the most common method for assessing MR severity. Accordingly, the aim of this study was to compare the results of echocardiography and cardiac MRI for assessing MR severity in a cohort of patients with HCM. MR severity was assessed in 53 patients using cardiac MRI by determining the mitral RV (MRV) and mitral RF (MRF). The results were graded according to thresholds recommended in current guidelines. MR severity assessed by echocardiography was graded by integrating indices of severity. Greater than mild MR, as assessed using echocardiography, was present in 22 patients (41.5%) with HCM and in none of the control patients (p = 0.001). In all, 31 patients (58.5%) had no more than mild MR. When MR severity was assessed using different methods, either moderate (kappa = 0.44, 95% confidence interval = 0.21–0.67), poor or no agreement was found between MRI-derived and echocardiography-derived grades. HCM patients with echocardiography-derived moderate and severe MR had similar median MRVs and MRFs (p = 0.59 and p = 0.11, respectively). In HCM patients, cardiac MRI and echocardiography were at most in modest agreement in assessing MR severity. Importantly, echocardiography-derived moderate and severe MR were not distinguishable by either MRV or MRF.

## Introduction

Mitral regurgitation (MR) remains one of the most common valvular heart diseases^1–3^, significantly affecting patients’ symptoms and long-term prognoses. MR is an important feature of hypertrophic cardiomyopathy (HCM), which is one of the factors responsible for heart failure symptoms (with shortness of breath being one of the most frequent clinical presentations) and the development of atrial fibrillation^4–7^. The presence and mechanisms of MR and concomitant abnormalities of the mitral valve apparatus affect which treatment options are chosen^5,8^. Thus, it is of paramount importance to obtain a reliable assessment of MR severity in HCM patients. However, although enormous progress has been made in non-invasive cardiac imaging, assessments of MR severity remain controversial, particularly in cases with functional regurgitation, which is the main type of MR in HCM^1,2,8–10^. Echocardiography is the most common method for assessing MR severity in a variety of populations, including patients with HCM. However, it has some limitations that render it prone to errors when used to grade MR severity in HCM patients^9,11^. Thus, using echocardiography to assess MR in this population presents an ongoing and persistent challenge. Cardiac magnetic resonance imaging (MRI) provides reliable estimates of ventricular and regurgitant volumes and is therefore considered a reference method that is particularly useful in doubtful or borderline cases^9^. Accordingly, we aimed to compare the results of echocardiography and MRI when used to assess MR severity in a cohort of patients with HCM.

## Results

### Patient selection and baseline characteristics

A total of 354 patients who were referred for cardiac MRI fulfilled the inclusion criteria during the analysed period. Among these, 154 patients were excluded based on the prescribed exclusion criteria (n = 37 had studies with artefacts and/or that were terminated before phase-contrast images were obtained; n = 32 had an equivocal diagnosis; n = 30 had a final diagnosis other than HCM; n = 23 received prior septal reduction therapies; n = 15 exhibited atrial fibrillation during echocardiography and/or an MRI scan; n = 5 studies were not completed due to severe claustrophobia, extreme obesity or the patient’s inability to lay flat; and n = 12 for other reasons). Among the remaining subjects (n = 180 and n = 20 HCM patients and controls, respectively), pulmonary flow data from 143 HCM patients (79.4%) and 15 individuals without HCM (75.0%) were available. Subsequently, we excluded those in whom time between the MRI study and echocardiography was greater than 7 days. These patients (n = 53) formed the final study population. The mean age of the study population was 49.7 ± 14.7 years, and 62.3% of the participants (n = 33) were men (Table 1). The median time between the MRI study and echocardiography was 1 day (IQR = 0–7 days). None of the patients underwent invasive procedures or had a change in medical treatment within this interval. All included patients remained clinically stable.

**Table 1:** Baseline characteristics of the study group and the control group.

Characteristic	Patient group	Control group	p
Mean age ± SD, years	49.7 ± 14.7	37.5 ± 17.8	0.009
Males, n (%)	33 (62.3%)	12 (80.0%)	0.24
Median time between the MRI study and echocardiography (IQR)	1 (0–7)	3 (1–4)	0.10
Median LVEDV (IQR), mL/m^2^	94.5 (87.3–102.0)	95.8 (83.2–102.8)	0.70
Median LVEF (IQR), %	64.2 (60.9–69.2)	64.0 (61.1–69.1)	0.87
Mean LVM ± SD, g/m^2^	89.6 ± 28.9	58.4 ± 11.8	< 0.0001
Median LVOT gradient (IQR), mmHg	30 (15–45)	9 (9–9)	0.0001
LVOT obstruction^a^, n (%)	29 (54.7%)	–	–

IQR, interquartile range; LVEDV, left ventricular end-diastolic volume; LVEF, left ventricular ejection fraction; LVM, left ventricular mass; LVOT, left ventricular outflow tract; MRI, magnetic resonance imaging; SD, standard deviation.

^a^Peak LVOT gradient greater than or equal to 30 mmHg.

More than mild MR, as assessed using echocardiography, was present in 22 patients (41.5%) with HCM (14 with moderate and 8 with severe regurgitation) and in none of the control patients (p = 0.001). The remaining 31 patients with HCM (58.5%) had no more than mild (mild, trivial or absent) MR.

### Comparison of MR severity between echocardiography and MRI using MRV

Moderate agreement was identified between echocardiography and MRI grades based on the MRV (kappa = 0.44, 95% confidence interval (CI) = 0.21–0.67, Table 2). Agreement was noted in 31 of 53 patients (58.5%) (i.e., both echocardiography and MRI indicated the same grade of MR). Only 2 of 8 patients (25.0%) with severe MR according to echocardiography also had severe MR according to MRI. Moreover, 1 patient (12.5%) with severe MR on echocardiography had mild MR on MRI. However, of 4 patients in whom MRI indicated severe MR (MRV ≥ 60 ml), 1 (25%) had mild MR on echocardiography (Table 2).

**Table 2:** Comparison between echocardiography-derived and MRI-derived mitral regurgitation grades.

	Mitral regurgitation severity by echocardiography			Total
	Mild	Moderate	Severe	
Mitral regurgitation severity by MRI				
Mild	21	5	1	27 (50.9%)
Moderate	9	8	5	22 (41.5%)
Severe	1	1	2	4 (7.6%)
Total	31 (58.5%)	14 (26.4%)	8 (15.1%)	53

Agreement was also poor when only severe and non-severe regurgitation (i.e., mild and moderate MR were combined) were considered (kappa = 0.26, 95% CI = − 0.10–0.62) or when patients were divided according to the presence of LVOT obstruction (peak gradient < 30 mmHg vs ≥ 30 mmHg or using a more specific but less sensitive cut-off, namely < 50 mmHg vs ≥ 50 mmHg). We have also assessed the agreement between echo and MRI in the identification of the presence of systolic anterior motion (SAM), and we have found that there was very good agreement between these two modalities (kappa = 0.84, 95% CI = 0.70–0.99). Out of 53 patients, a discrepancy between echo and MRI was found only in 4 (7.5%). After exclusion of these patients, there remained only moderate agreement between echo and MRI in grading mitral regurgitation (kappa = 0.46, 95% CI 0.22–0.69). We have investigated also whether the LV maximal wall thickness in the basal antero-septal segment correlated with the degree of MR. There was a weak but positive association between these variables (rho = 0.39, p = 0.004).

In the majority of patients with mild MR according to echocardiography (67.7%, 21 out of 31), MRI confirmed mild regurgitation (Table 2).

### Comparison of MR severity between echocardiography and MRI using the MRF

For the MRF-based categories, there was no agreement with echocardiography-based grading (kappa = 0.05, 95% CI = − 0.07–0.18). No patients had an MRF ≥ 50%, which was the threshold used to define severe MR.

### Comparison of MRI-derived MRV and MRF with echocardiography grades

**Figure 1:**
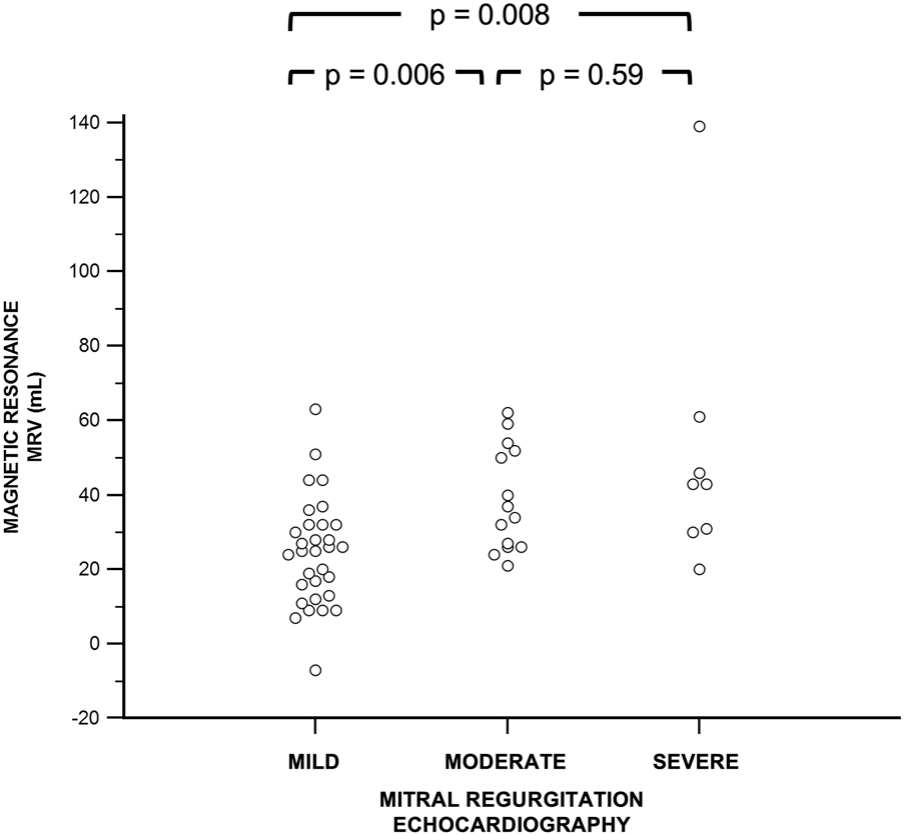
Comparison of mitral regurgitation volume (MRV) among patients with mild, moderate, and severe mitral regurgitation assessed by echocardiography.

The results of the MRV calculations in patients with mild, moderate, or severe MR according to echocardiography are shown in Fig. 1. Although the Kruskal–Wallis test revealed that there were statistically significant differences in MRV (p = 0.003), in pairwise analyses, there were no differences in MRV between patients with moderate and severe MR assessed using echocardiography (Fig. 1). HCM patients with echocardiography-derived moderate MR had median MRV values similar to those found in patients with echocardiography-derived severe MR (Fig. 1).

**Figure 2:**
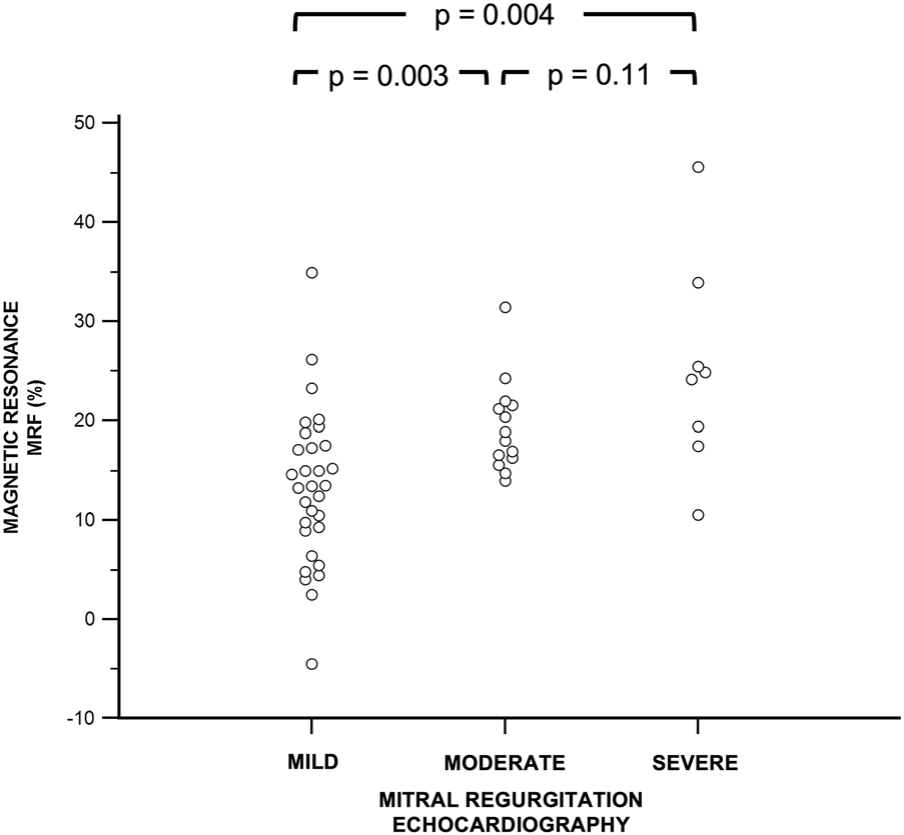
Comparison of mitral regurgitation fraction (MRF) among patients with mild, moderate, and severe mitral regurgitation assessed by echocardiography.

The results of the comparison of MRF mirrored the results of the same comparison of MRV values (Fig. 2). There were no differences in MRF between patients with echocardiography-derived moderate and severe MR (Fig. 2).

### Comparison of MRV and MRF in the assessment of MR severity

**Figure 3:**
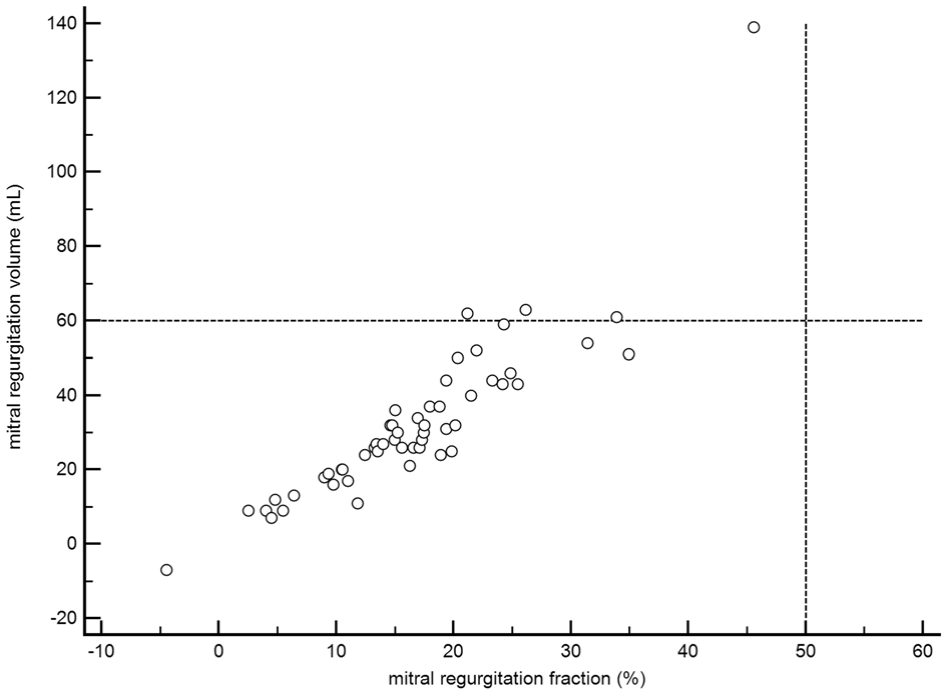
Correlation between the mitral regurgitation fraction (MRF) and mitral regurgitation volume (MRV).

No patient fulfilled the criteria for severe MR based on the MRF (MRF ≥ 50%, Fig. 3). However, 4 patients had severe MR when it was defined as an MRV ≥ 60 ml. In these patients, the MRF ranged from 21.2 to 45.5%, fulfilling the criteria for either moderate (n = 2) or mild MR (n = 2). An excellent correlation was found between the MRV and MRF (rho = 0.91, 95% CI = 0.85–0.95, p < 0.0001; Fig. 3).

### Mitral regurgitation assessment using MRI in the control group

In all controls, echocardiography indicated mild MR. The mean MRV was 19.5 ± 7.1 ml. The mean MRF was 10.3 ± 3.2%.

## Discussion

The main finding of our study is that in HCM patients, cardiac MRI and echocardiography showed at most modest agreement when used to assess MR severity. Importantly, echocardiography-derived moderate MR and severe MR were not distinguishable regardless of the MRI-derived MRV or MRF quantification method used. Additionally, in a substantial proportion of the patients (75%) with severe MR on echocardiography, the MRI-based MRV was below the defined threshold for severe MR. Conversely, some patients with mild MR according to echocardiography had severe MR according to MRI.

In patients with valvular regurgitation, it is always crucial to identify severe cases of regurgitation. For the vast majority of patients, echocardiography is the first-line method for assessing MR severity. However, MRI-based MRV is considered the reference standard for MRV quantification in studies comparing the results of using echocardiography and MRI to assess MR severity^12–16^. In our study, there was no difference in MRI-calculated MRV between patients with moderate and severe MR graded using echocardiography. In other words, patients with moderate and severe MR on echocardiography were indistinguishable when MRI-derived MRV was used. Moreover, when we expressed MRI-derived MR severity as MRF, similar results were obtained. These findings may be clinically relevant since significant LVOT obstruction with concomitant severe MR leads to extension of septal reduction therapy from isolated myectomy to myectomy with concomitant mitral valve and mitral apparatus surgery.

Apart from being able to adequately identify severe MR, being able to unequivocally diagnose mild regurgitation is also particularly important, which allows the physician to refrain from further diagnostic tests, and mild regurgitation is associated with good long-term prognoses. In this study, in most patients with mild MR on echocardiography, MRI also indicated mild regurgitation. Of 31 individuals in whom echocardiography indicated mild MR, MRI indicated a different grade (moderate MR) in 9 patients (29%) and a severe grade in one additional patient (3.2%).

These findings are consistent with those presented in previous studies indicating a discrepancy between the results of using echocardiography and MRI to assess MR severity in various patient populations^12–15^. However, quantifying the MRV using echocardiography is challenging in HCM patients, and these patients were excluded from previous studies^13^, leading to questions about the accuracy of echocardiography (the first-line diagnostic technique, as mentioned above) when used to assess MR severity in HCM patients. Ideally, a comparison of MRI-derived and echocardiography-derived MRV should provide the data needed to answer this question. We did not compare MRV between echocardiography and MRI. Instead, the severity of MR was compared using mild, moderate, and severe categories. Echocardiography grades were defined based on an integrative approach, while cardiac MRI grades were based on calculated MRV or MRF. However, considering the lack of uniform MRI-based thresholds for MR grading, we used a cut-off that is commonly used for echocardiography that has also been used in previous investigations comparing these two imaging modalities^13^. Our goal was to determine whether high-quality, standard-of-care echocardiography can provide a reliable assessment of the severity of MR that is comparable to quantitative assessments performed using MRI in HCM patients tested under normal, real-life conditions similar to those found in clinics around the world. To the best of our knowledge, no previous studies have quantitatively assessed MR using echocardiography in this challenging population. Conversely, there is evidence indicating that performing volumetric (MRV) assessments using echocardiography in HCM patients is very demanding because patients with HCM were excluded form a landmark study by Uretsky et al.^13^. However, in the same paper, Uretsky et al. demonstrated that the discrepancy between echocardiography-based and MRI-based estimates of MR severity was present independent of whether a categorical (i.e., mild, moderate, and severe) or quantitative (i.e., a comparison of MRVs) approach was used^13^. Thus, comparing MRVs is not necessary to assess the level of agreement between cardiac MRI and echocardiography when used to assess MR severity. The comparison conducted using severity grades performed well and also presents one more advantage in that in a clinical scenario, this is the severity grade that determines whether intervention or conservative treatment is used according to current guidelines^1,2,9^.

Previous studies have sought to determine the MRI threshold for the MRF that best reflects MR regurgitation severity compared to echocardiography (i.e., results in the best agreement with echocardiography)^17^. However, when assessing MR severity, substantial discordance in the results obtained using echocardiography and MRI has been demonstrated^12–15^. Additionally, MRI-derived regurgitation severity was more highly correlated with postoperative LV remodelling, indicating that this method (cardiac MRI) better reflects true volume overload^13^. Thus, defining an MRI threshold that best correlates with echocardiography is not currently useful.

### Limitations

The main limitation of our study is the dynamic nature of LVOT obstruction, which can potentially cause variations in MR severity. Although none of the patients in this study underwent invasive procedures or had a change in medical treatment between echocardiography and MRI, fluctuations in LVOT gradient can per se impact the severity of SAM-related MR. Performing two studies (echocardiography and MRI) during one day or immediately performing one study after completing the other does not solve this issue because variations in LVOT gradients are attributable not only to loading conditions, medications used, and prandial status but also to beat-to-beat and respiratory changes. The finding that the exclusion of patients with discrepancies (echo vs. MRI) in assessing the presence of SAM did not improve the agreement between the two modalities may be due to the fact that despite the presence of SAM, a variable degree of LVOT obstruction was present at the time of echo and MRI. This, as mentioned above, is an inherent limitation of studies assessing the dynamic nature of LVOT obstruction in HCM patients.

According to the current guidelines, a final assessment of MR severity is made by integrating Doppler and morphological information^1,2,9,18^. Estimating MRV using 2-dimensional echocardiography in patients with LVOT obstruction remains challenging because the flow convergence method is less accurate in the eccentric jets typically seen in patients with obstructive HCM^12,19^. The SAM-related regurgitant jet observed in HCM presents a number of characteristics, such as being noncircular, eccentric, and nonholosystolic, that limit the accuracy of performing a quantitative analysis using echocardiography. Additionally, the presence of additional criteria for severe MR, such as a dilated left atrium, is not useful for assessing mitral regurgitation severity. In HCM patients, the size of the left atrium may be abnormal not only due to MR but also as a result of diastolic dysfunction and LVOT obstruction^20^. Quantifying MRV with 3-dimensional echocardiography, which has been shown to overcome the handicap of 2-dimensional echocardiography for quantifying MRV, should be explored in HCM patients in future studies. Such findings should be compared with MRI findings calculated via various techniques to identify the most suitable method for quantifying the MRV in this challenging population^21–23^. The echocardiographic studies herein were performed mainly for clinical purposes and were not conducted using sophisticated research protocols focused specifically on mitral regurgitation. However, as mentioned above, all studies were performed using high-quality cardiovascular ultrasound systems by experienced physicians who made the decision of whether to use and how to interpret various components of an integrative approach to define valve regurgitation severity.

Although an MRV ≥ 60 ml or an MRF ≥ 50% was used to define severe MR, in accordance with current guidelines, severe MR should also be recognized at lower thresholds (i.e., 45–59 ml for MRV and for 40–49% for MRF) provided that additional criteria for severe MR are present or the orifice is elliptical^9^. Additionally, the MRV for severe regurgitation may be lower under low flow conditions^9^ and in some patients with HCM and a small left ventricular cavity^24^.

We do not have full data on heart rate at the moment of echocardiography to compare it with heart rate at the moment of MRI. This may limit the findings of our study since heart rate can influence the severity of MR.

A weak but positive association between LV maximal wall thickness in the basal antero-septal segment and the MRV was observed (rho = 0.39, p = 0.004). This confirms the notion that the localization and pattern of hypertrophy impacts the severity of MR. Additionally, it has been shown that the spectrum of leaflet length and mobility that affects subaortic obstruction also influences mitral regurgitation in patients with SAM (29). We believe that more extensive deliberating on this topic is beyond the scope of the present analysis and should be adequately addressed in future prospective studies focused particularly on this topic.

Finally, a truly accurate reference method for assessing MR severity has not yet been established. No consensus is currently available for MR assessments performed with either echocardiography or MRI. Regarding echocardiography, in a recent update of the American College of Cardiology/American Heart Association guidelines, it was recommended that a 60-ml threshold should be used for both severe primary and secondary MR, while recent European Society of Cardiology guidelines distinguish between severe primary and secondary MR in terms of MRV (60 ml and 30 ml, respectively)^1,2^.

Some authors advocate that the extent of left ventricular reverse remodelling following surgical intervention should provide a validation of preoperative MR severity^13^. This approach, however, would be difficult to perform in HCM patients because mitral valve repair or replacement is performed simultaneously with surgical myectomy in the vast majority of such cases as a result of severe left ventricular outflow tract obstruction, and relief of this obstruction results in an increase in left ventricular size^25–27^. Nevertheless, future studies aimed at comparing outcomes among different methods of assessing MR severity are strongly desirable.

## Methods

### Study population

We prospectively recruited consecutive patients with HCM or suspected HCM who were referred for cardiac MRI between the beginning of January 2015 and the end of January 2017 in a tertiary care centre for HCM patients. Herein, we present a retrospective analysis performed on a prospectively included cohort of HCM patients. Patients with a history of any septal reduction therapy or mitral valve repair or replacement were excluded. Additionally, to obtain the most reliable data concerning ventricular and valve function, patients with atrial fibrillation or frequent ventricular or supraventricular arrhythmias during echocardiography and/or MRI scanning were excluded. Patients in whom a diagnosis of HCM was not confirmed (e.g., when Fabry disease or cardiac amyloidosis was the final diagnosis) or was equivocal (e.g., differential diagnosis between HCM versus hypertensive heart disease or athlete’s heart) were not considered^28,29^. All patients who had more than trivial/mild aortic or pulmonary regurgitation defined as phase-contrast-derived regurgitation fraction ≤ 10% were excluded. The control group consisted of patients referred for cardiac MRI with suspected HCM in whom HCM was eventually excluded. The control group consisted of asymptomatic patients referred for cardiac magnetic resonance imaging (MRI) due to suspicion of hypertrophic cardiomyopathy (HCM). The suspicion of cardiomyopathy was based either on a family history of hypertrophic cardiomyopathy (in such cases, performing cardiac MRI was a part of the screening of the relatives of probands diagnosed with HCM) or on echocardiography indicating left ventricular hypertrophy. However, as we have previously shown in HCM patients^30^, echocardiography may overestimate the maximal left ventricular wall thickness, leading to a false diagnosis of HCM (i.e., echocardiography indicates left ventricular hypertrophy, while the true left ventricular wall thickness measured with cardiac MRI is within the normal limit). In summary, the control group comprised individuals without symptoms in whom HCM or other cardiac diseases were excluded based on cardiac MRI. The study protocol conformed to the ethical guidelines of the 1975 Declaration of Helsinki and was approved by the local ethics committee of the National Institute of Cardiology, Warsaw, Poland. All patients provided written informed consent.

### MRI

All cardiac MRI studies were performed on a 1.5 T scanner (Avanto/Avanto^fit^, Siemens, Erlangen, Germany). Left ventricular (LV) volumes (end-diastolic volume, LVEDV; end-systolic volume, LVESV; stroke volume, LVSV), mass (LVM), and ejection fraction (LVEF) were calculated on the basis of a stack of short-axis cine images (balanced steady state free-precession, ECG triggered, breath-hold acquisition) that covered the ventricles from the base to the apex (typical parameters: 25 phases, echo time 1.2 ms, repetition time 33–54 ms, echo spacing 2.7 ms, flip angle 64°–79°, slice thickness 8 mm, and gap 2 mm). The manual delineation of epicardial and endocardial contours at end-diastole and end-systole was performed using dedicated software (QMass 7.6, Medis, Leiden, Netherlands). The LV segmentation algorithm included the papillary muscles and trabeculae in the blood pool but excluded them from LVM^31^, which is the standard method used in most MRI studies, including studies comparing MRI and echocardiography in various valvular heart diseases.

Aortic and pulmonary flow data were obtained using a breath-hold, phase contrast-sensitive sequence (typical parameters: echo time 2.5 ms, repetition time 30–47 ms, flip angle 30°, and section thickness 5 mm). Images were obtained in a plane perpendicular to the vessel wall at the mid-point of the main pulmonary artery, providing 30 phase and magnitude images per cardiac cycle. Velocity encoding was adjusted to avoid aliasing. Phase contrast data were analysed with a semiautomatic vessel edge-detection algorithm with operator correction using dedicated software (QFlow 5.6, Medis, Leiden, the Netherlands). Additionally, to confirm that background phase errors did not significantly affect the results, we used this software to perform corrections. We employed main pulmonary artery (MPA) flow data to calculate MRV. This approach (MRV = LVSV − pulmonary artery forward flow) has been recommended as an alternative to aortic flow-based calculations^9^. Pulmonary flow-based calculations are not affected by turbulent flow caused by LV outflow tract (LVOT) obstruction and therefore provide more reliable MRV estimates. Additionally, we expressed MR severity not only as absolute numbers (MRV) but also as a relative measure compared to LVSV (mitral regurgitation fraction: MRF). Hence, we used the following 2 different methods to quantify MR:

- Method 1: MRV = LVSV − MPA.
- Method 2: MRF = $$\frac{{{\text{LVSV}} - {\text{MPA}}}}{{{\text{LVSV}}}} \times 100\%$$

All cardiac MRI studies and analyses were performed by physicians with more than 10 years of experience in assessing HCM patients. These physicians have Level 3 Certificates from the European Association of Cardiovascular Imaging. Intra- and inter-observer variability in both cine and PC data analyses have previously been reported^31–33^.

We used the following thresholds to grade MR severity according to the current guidelines^9^:

- Based on MRV: mild (< 30 ml), moderate (30–59 ml), and severe ≥ 60 ml, and
- Based on MRF: mild (< 30%), moderate (30–49%), and severe ≥  50%.

### Echocardiography

All patients underwent high-quality standard-of-care echocardiography studies that were performed using commercially available systems (GE Medical Systems Vivid 7 or 9 with a 2.5 MHz transducer). MR was graded as mild, moderate or severe by integrating the indices of severity^1,2,9,18^. First, when the MR jet met the visual assessment criteria for classification as mild, regurgitation was classified as mild^9^. Second, patients with presumably severe regurgitation were identified. In doubtful cases, multiparametric analysis was applied. Which components of the integrated approach (valve morphology, colour Doppler interrogation of the MR jet, continuous-wave Doppler signal of the regurgitant jet, vena contracta width, flow convergence zone, systolic pulmonary vein flow reversal, and mitral inflow velocities) were used was left to the discretion of the experienced physician performing the study. The following criteria were employed as markers of mild MR: small, narrow central jet, vena contracta width ≤ 3 mm, PISA radius absent or ≤ 3 mm at Nyquist 30–40 cm/s, mitral A wave dominant inflow, and soft or incomplete jet by continuous wave Doppler (7). On the other hand, vena contracta width ≥ 7 mm, PISA radius ≥ 10 mm at Nyquist 30–40 cm/s, large jet > 50% of the left atrium area, and pulmonary vein systolic flow reversal were considered specific for severe regurgitation (7). In our centre, which is a tertiary reference hospital for HCM patients, all echocardiography studies performed in patients with HCM or the suspicion of HCM are performed by physicians with adequate experience in assessing patients with HCM, including experience in performing such evaluations prior to, during, and after septal reduction therapies. In doubtful cases, a senior consultant cardiologist with over 30 years of experience in performing echocardiography studies in HCM patients was consulted. Additionally, as part of the routine procedure for performing ultrasound evaluations in patients with HCM, the presence of an LVOT gradient at rest and during provocation was assessed, and a peak gradient of 30 mmHg or higher was taken to indicate the presence of LVOT obstruction^34^.

### Statistical analysis

Continuous data were assessed for a normal distribution using the Kolmogorov–Smirnov test and are expressed as either the means ± standard deviation (SD) or the medians with an interquartile range (IQR), as appropriate. Depending on the distribution, continuous data were compared using Student’s t-test or the Mann–Whitney test. Categorical data are presented as frequency percentages and were compared using either the Fisher exact test or the Chi-square test. The Kruskal–Wallis test was used to compare MRVs and MRFs among patients with mild, moderate, and severe MR as assessed by echocardiography. For pairwise comparisons, Bonferroni correction was used to determine statistical significance (0.05 ÷ 3 = 0.017). To analyse the degree of agreement between MRI and echocardiography evaluations, a weighted kappa test with quadratic weights was used. The Spearman coefficient of correlation (rho) was used to assess correlations between non-normally distributed variables. All statistical analyses were performed using MedCalc 19.1.3 software (MedCalc, Ostend, Belgium).

## Conclusions

In HCM patients, intermodality agreement (MRI vs. echocardiography) was at most modest for grading the severity of mitral regurgitation. Importantly, echocardiography-derived moderate and severe MR were not distinguishable based on either MRV or MRF.

## Data Availability

The datasets generated and/or analysed during the current study are available from the corresponding author on reasonable request.

## References

[1] Nishimura RA (2017). 2017 AHA/ACC focused update of the 2014 AHA/ACC guideline for the management of patients with valvular heart disease: a report of the American College of Cardiology/American Heart Association Task Force on Clinical Practice Guidelines. Circulation.

[2] Baumgartner H (2017). 2017 ESC/EACTS Guidelines for the management of valvular heart disease. Eur. Heart J..

[3] Otto CM (2020). ACC/AHA guideline for the management of patients with valvular heart disease: executive summary—a report of the American College of Cardiology/American Heart Association Joint Committee on Clinical Practice Guidelines. Circulation.

[4] Moon I (2020). Trends of the prevalence and incidence of hypertrophic cardiomyopathy in Korea: a nationwide population-based cohort study. PLoS ONE.

[5] Elliott PM (2014). 2014 ESC Guidelines on diagnosis and management of hypertrophic cardiomyopathy: the task force for the diagnosis and management of hypertrophic cardiomyopathy of the European Society of Cardiology (ESC). Eur. Heart J..

[6] Gersh BJ (2011). ACCF/AHA guideline for the diagnosis and treatment of hypertrophic cardiomyopathy: a report of the American College of Cardiology Foundation/American Heart Association task force on practice guidelines. Developed in collaboration with the American Association for Thoracic Surgery, American Society of Echocardiography, American Society of Nuclear Cardiology, Heart Failure Society of America, Heart Rhythm Society, Society for Cardiovascular Angiography and Interventions, and Society of Thoracic Surgeons. J. Am. Coll. Cardiol..

[7] Ommen SR (2020). 2020 AHA/ACC guideline for the diagnosis and treatment of patients with hypertrophic cardiomyopathy: executive summary: a report of the American College of Cardiology/American Heart Association Joint Committee on Clinical Practice Guidelines. Circulation.

[8] Hong JH (2016). Mitral regurgitation in patients with hypertrophic obstructive cardiomyopathy: implications for concomitant valve procedures. J. Am. Coll. Cardiol..

[9] Zoghbi WA (2017). Recommendations for noninvasive evaluation of native valvular regurgitation: a report from the American Society of echocardiography developed in collaboration with the society for cardiovascular magnetic resonance. J. Am. Soc. Echocardiogr..

[10] Lancellotti P (2013). Recommendations for the echocardiographic assessment of native valvular regurgitation: an executive summary from the European Association of Cardiovascular Imaging. Eur. Heart J. Cardiovasc. Imaging.

[11] Lancellotti P (2010). European Association of Echocardiography recommendations for the assessment of valvular regurgitation: part 2: mitral and tricuspid regurgitation (native valve disease). Eur. J. Echocardiogr..

[12] Krieger EV, Lee J, Branch KR, Hamilton-Craig C (2016). Quantitation of mitral regurgitation with cardiac magnetic resonance imaging: a systematic review. Heart.

[13] Uretsky S (2015). Discordance between echocardiography and MRI in the assessment of mitral regurgitation severity: a prospective multicenter trial. J. Am. Coll. Cardiol..

[14] Lopez-Mattei JC (2016). Comparative assessment of mitral regurgitation severity by transthoracic echocardiography and cardiac magnetic resonance using an integrative and quantitative approach. Am. J. Cardiol..

[15] Cawley PJ (2013). Prospective comparison of valve regurgitation quantitation by cardiac magnetic resonance imaging and transthoracic echocardiography. Circ. Cardiovasc. Imaging.

[16] Penicka M (2017). Prognostic implications of magnetic resonance—derived quantification in asymptomatic patients with organic mitral regurgitation: comparison with Doppler echocardiography-derived integrative approach. Circulation.

[17] Gelfand EV (2006). Severity of mitral and aortic regurgitation as assessed by cardiovascular magnetic resonance: optimizing correlation with Doppler echocardiography. J. Cardiovasc. Magn. Reson..

[18] Lancellotti P (2010). European Association of Echocardiography recommendations for the assessment of valvular regurgitation: part 2—mitral and tricuspid regurgitation (native valve disease). Eur. J. Echocardiogr..

[19] Grayburn PA, Weissman NJ, Zamorano JL (2012). Quantitation of mitral regurgitation. Circulation.

[20] Yang H (2005). Enlarged left atrial volume in hypertrophic cardiomyopathy: a marker for disease severity. J. Am. Soc. Echocardiogr..

[21] Thavendiranathan P (2013). Quantification of chronic functional mitral regurgitation by automated 3-dimensional peak and integrated proximal isovelocity surface area and stroke volume techniques using real-time 3-dimensional volume color Doppler echocardiography: in vitro and clinical validation. Circ. Cardiovasc. Imaging.

[22] Shanks M (2010). Quantitative assessment of mitral regurgitation: comparison between three-dimensional transesophageal echocardiography and magnetic resonance imaging. Circ. Cardiovasc. Imaging.

[23] Hamada S (2012). Comparison of accuracy of mitral valve regurgitation volume determined by three-dimensional transesophageal echocardiography versus cardiac magnetic resonance imaging. Am. J. Cardiol..

[24] Rawlins J, Bhan A, Sharma S (2009). Left ventricular hypertrophy in athletes. Eur. J. Echocardiogr..

[25] Dabrowski M (2012). An assessment of regression of left ventricular hypertrophy following alcohol ablation of the interventricular septum in patients with hypertrophic cardiomyopathy with left ventricular outflow tract obstruction. Kardiol. Pol..

[26] Rivera S (2003). Left ventricular remodeling in patients with hypertrophic obstructive cardiomyopathy treated with percutaneous alcohol septal ablation: an echocardiographic study. Rev. Esp. Cardiol..

[27] Chen YZ (2015). Biventricular reverse remodeling after successful alcohol septal ablation for obstructive hypertrophic cardiomyopathy. Am. J. Cardiol..

[28] Jiang M (2018). The significance of interstitial fibrosis on left ventricular function in hypertensive versus hypertrophic cardiomyopathy. Sci. Rep..

[29] Huang S (2020). Left ventricular global function index by magnetic resonance imaging—a novel marker for differentiating cardiac amyloidosis from hypertrophic cardiomyopathy. Sci. Rep..

[30] Śpiewak M (2020). Impact of cardiac magnetic resonance on the diagnosis of hypertrophic cardiomyopathy—a 10-year experience with over 1000 patients. Eur. Radiol..

[31] Śpiewak M (2017). Quantification of mitral regurgitation in patients with hypertrophic cardiomyopathy using aortic and pulmonary flow data: impacts of left ventricular outflow tract obstruction and different left ventricular segmentation methods. J. Cardiovasc. Magn. Reson..

[32] Śpiewak M (2012). Repaired tetralogy of Fallot: ratio of right ventricular volume to left ventricular volume as a marker of right ventricular dilatation. Radiology.

[33] Mazurkiewicz Ł (2017). Systolic myocardial volume gain in dilated, hypertrophied and normal heart CMR study. Clin. Radiol..

[34] Elliott PM (2014). 2014 ESC guidelines on diagnosis and management of hypertrophic cardiomyopathy: the task force for the diagnosis and management of hypertrophic cardiomyopathy of the European Society of Cardiology (ESC). Eur Heart J.

